# Opc Expression, LPS Immunotype Switch and Pilin Conversion Contribute to Serum Resistance of Unencapsulated Meningococci

**DOI:** 10.1371/journal.pone.0045132

**Published:** 2012-09-20

**Authors:** Kerstin Hubert, Marie-Christin Pawlik, Heike Claus, Hanna Jarva, Seppo Meri, Ulrich Vogel

**Affiliations:** 1 University of Würzburg, Institute for Hygiene and Microbiology, Würzburg, Germany; 2 Haartman Institute, Helsinki, Finland; University of Kentucky College of Medicine, United States of America

## Abstract

*Neisseria meningitidis* employs polysaccharides and outer membrane proteins to cope with human serum complement attack. To screen for factors influencing serum resistance, an assay was developed based on a colorimetric serum bactericidal assay. The screening used a genetically modified sequence type (ST)-41/44 clonal complex (cc) strain lacking LPS sialylation, polysaccharide capsule, the factor H binding protein (fHbp) and MutS, a protein of the DNA repair mechanism. After killing of >99.9% of the bacterial cells by serum treatment, the colorimetric assay was used to screen 1000 colonies, of which 35 showed enhanced serum resistance. Three mutant classes were identified. In the first class of mutants, enhanced expression of Opc was identified. Opc expression was associated with vitronectin binding and reduced membrane attack complex deposition confirming recent observations. Lipopolysaccharide (LPS) immunotype switch from immunotype L3 to L8/L1 by *lgtA* and *lgtC* phase variation represented the second class. Isogenic mutant analysis demonstrated that in ST-41/44 cc strains the L8/L1 immunotype was more serum resistant than the L3 immunotype. Consecutive analysis revealed that the immunotypes L8 and L1 were frequently observed in ST-41/44 cc isolates from both carriage and disease. Immunotype switch to L8/L1 is therefore suggested to contribute to the adaptive capacity of this meningococcal lineage. The third mutant class displayed a *pilE* allelic exchange associated with enhanced autoaggregation. The mutation of the C terminal hypervariable region D of PilE included a residue previously associated with increased pilus bundle formation. We suggest that autoaggregation reduced the surface area accessible to serum complement and protected from killing. The study highlights the ability of meningococci to adapt to environmental stress by phase variation and intrachromosomal recombination affecting subcapsular antigens.

## Introduction

*Neisseria meningitidis*, a Gram-negative bacterium, requires resistance to human serum complement during invasive spread within the human bloodstream [1]. The search for neisserial surface factors interfering with human complement has recently been a major focus of research. The polysaccharide capsule contributes significantly to meningococcal serum resistance [1], [2] and unencapsulated meningococci rarely cause invasive meningococcal disease [3], but also subcapsular proteins affect the activation of complement. Proteins binding complement regulators have been identified by an approach now referred to as reverse vaccinology [4]. Genome sequencing, prediction of membrane proteins, and consecutive screening for proteins eliciting bactericidal antibodies led to the identification of the factor H (fH) binding protein fHbp [5] and the neisserial heparin binding antigen (NHBA) [6]. Both proteins, by binding of fH and heparin, respectively, regulate complement activation on the meningococcal surface [6], [7]. Studies with fHbp mutants showing residual fH binding led to the discovery of NspA as a fH ligand [8]. In addition, the meningococcal adhesin OpcA binds vitronectin [9]. Binding of vitronectin was suggested to interfere with membrane attack complex insertion in *Haemophilus influenzae* [10]. The meningococcal Msf and Opc proteins were reported to contribute to serum resistance by this means [11].

Genome wide screening of insertion libraries highlighted the dominant role of capsule and LPS [12]. Signature tagged mutagenesis has been used to study genes essential for survival in the infant rat model, again revealing the importance of capsule and LPS, but also of a couple of other factors including housekeeping enzymes [13]. In this report we developed a screen to identify single colonies with enhanced serum resistance. The method is an adaptation of a colorimetric serum bactericidal assay introduced by Rodriguez et al. [14], [15], which was designed for high throughput screening of sera. After serum stress, surviving bacteria metabolize glucose and the resulting acid production is visualized by a pH indicator dye. The assay was conducted with a mutant lacking capsule expression, fHbp expression and LPS sialylation to exclude dominant complement defense mechanisms. Furthermore, the *mutS* gene encoding a protein of the mismatch repair apparatus was mutated with the intention to enhance mutation and phase variation [16]. Phase variation is considered an important factor for adaptation of meningococci to the environment with 65 potentially phase variable genes [17], which, however, have not all been experimentally verified [18].

In the present study, a representative meningococcal strain of the ST-41/44 clonal complex (cc) of meningococci was analyzed. The ST-41/44 cc of meningococci accounts for a large proportion of serogroup B meningococcal disease worldwide including epidemic waves and outbreaks [19]–[21]. Despite its importance, genomes of this lineage have become publicly available only recently [22]. An outer membrane vesicle vaccine against ST-41/44 cc was used in New Zealand to combat a meningococcus B epidemic [23]. We used a strain from an outbreak in Western Germany, which according to available typing data very much resembles the New Zealand outbreak strain and was susceptible to antibodies elicited by the New Zealand outer membrane vesicle vaccine [21].

Using a colorimetric screening assay three mutant classes with elevated serum resistance were identified. Detailed analysis of each mutant class revealed a contribution of Opc expression, LPS immunotype switch and PilE variation to serum resistance in the absence of a capsule and fHbp. The paper reveals the potential of the screening assay for the analysis of bacterial adaptation to environmental stress. The findings elucidate the contribution of phase variation and intrachromosomal recombination to meningococcal host adaptation.

## Results

### Selection of strains

Serogroup B strain DE9686 (ST-41/44 cc) was genetically engineered to inactivate the capsule polysaccharide synthesis, LPS sialylation and expression of fHbp. In addition, the *mutS* gene, which encodes a protein involved in mismatch repair, was mutated to enhance the mutation and phase variation rate. In comparison to DE9686 *siaD- lst- fhbp*- with a spontaneous frequency of mutation to a rifampicin resistant phenotype of 1,42×10^−6^ (mean of *n* = 4, range: 5,0×10^−6^ to 3,5×10^−7^), the *mutS*- variant mutated with a 100-fold higher rate of 1,2×10^−4^ (mean of *n* = 4, range 4,4×10^−4^ to 9,1×10^−6^). Enhanced phase variation was elucidated by analysis of the poly-C stretch of the *opcA* promoter. Comparative sequencing of 89 randomly selected colonies from five independent experiments revealed that in the parental strain no phase variation occurred in the homopolymeric tract. In contrast, the *mutS*- strain showed phase variation in three of five experiments analyzing a total of 88 colonies. This finding confirmed that besides the higher mutation rate to rifampicin resistance, also phase variation was enhanced in the *mutS* mutant. The *mutS* mutant was subsequently used in the screening assay.

### Screening for serum resistant mutants using a colorimetric assay

**Figure 1:**
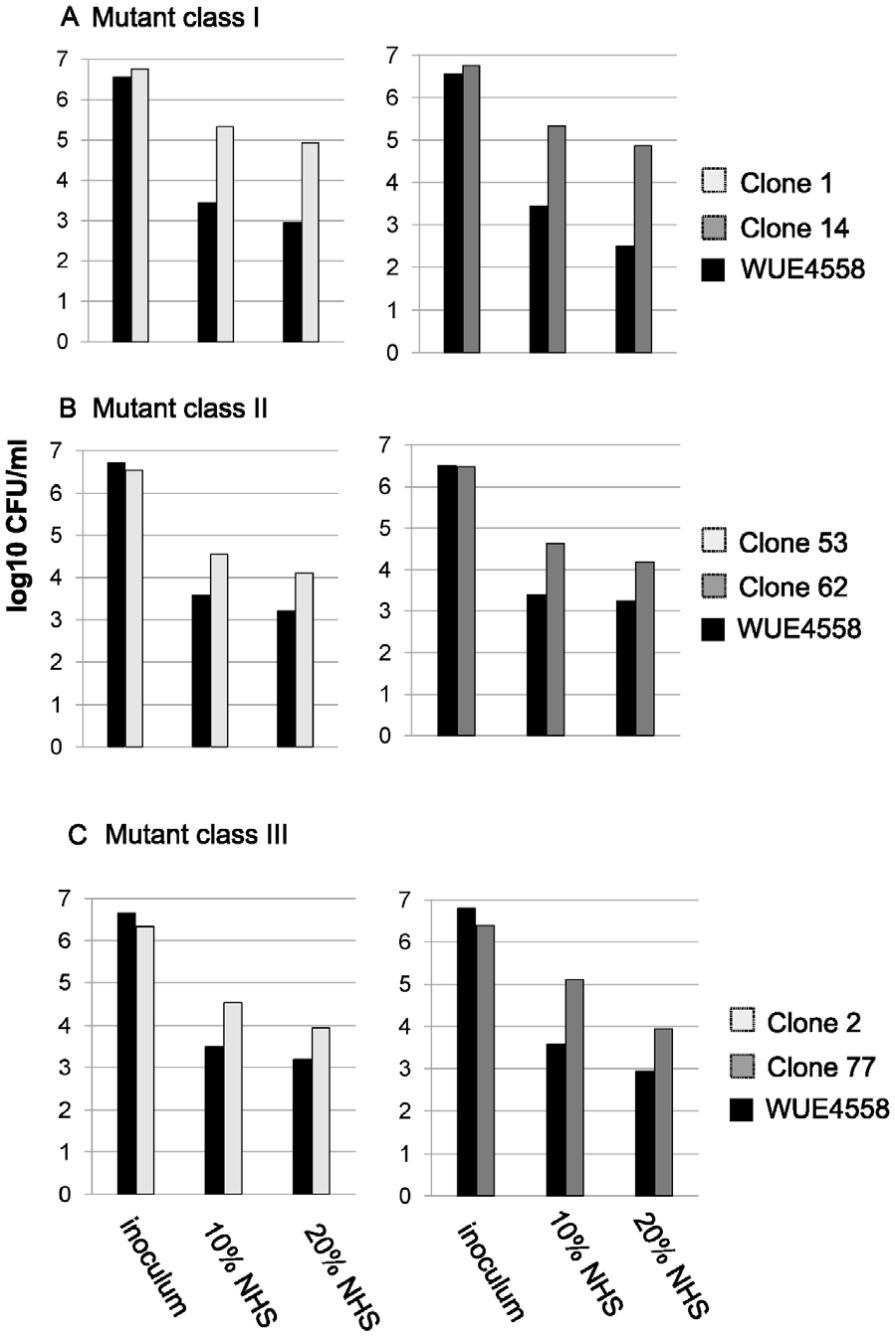
Serum killing assay of representative clones belonging to mutant classes I through III. The data display means of two to three independent experiments. (A) Clones 1 and 14 represent mutant class I overexpressing the Opc protein (n = 31); (B) Clones 53 and 62 (mutant class II, n = 2) displayed altered LPS phenotype compared to the parental strain WUE4558; (C) Clones 2 and 77 showed pilin variation (mutant class III, n = 2).

Bacterial suspensions of the DE9686 derivative with the genotype *siaD*- *lst*- *fhbp*- *mutS*-, which will be referred to as WUE4558 in the following, were treated with 10% normal human serum (NHS) for 30 min to kill 99.9% to 99.99% of the population and to select serum resistant variants. Individual colonies were again subjected to 10% NHS for 30 min in a 96-well microtiter plate format as described in Materials and Methods. Increased survival was indicated by colour change of the medium determined by visual inspection and OD reads of the microtiter plates. Variants with increased survival were rescreened by the same procedure. Of 1000 colonies, 90 were identified as serum resistant by the initial screening round; 35 of 90 were confirmed by repeated analysis in the screening assay at 10% NHS. Six clones with confirmed increased resistance in the colorimetric assay were selected for validation in a standard serum killing assay with plating of serial dilutions of bacterial suspensions. The serum killing assay confirmed the altered serum resistance phenotype with >10-fold increased survival (Fig. 1). The clones were selected from each mutant class described below, i.e. 2 of 31 clones from mutant class I, 2 of 2 clones from mutant class II, and 2 of 2 clones from mutant class III.

In order to assess the impact of *mutS* mutation on the number of serum resistant variants, 350 colonies of strain WUE4300 (*siaD*- *lst*- *fhbp*-) were obtained after serum treatment as described above and were then screened in the colorimetric assay. WUE4300 differs from WUE4558 in that the *mutS* gene was not inactivated. Increased serum resistance was confirmed in eleven of 350 colonies (3.1%). This proportion was not different from the screen with the *mutS* mutant (35 of 1000 colonies; 3.5%), suggesting that *mutS* mutation unexpectedly did not quantitatively add to the success of the screen despite the fact that *mutS* mutation increased the mutation and phase variation rate.

### Mutant class I: Increased expression of Opc in the majority of resistant colonies

**Figure 2:**
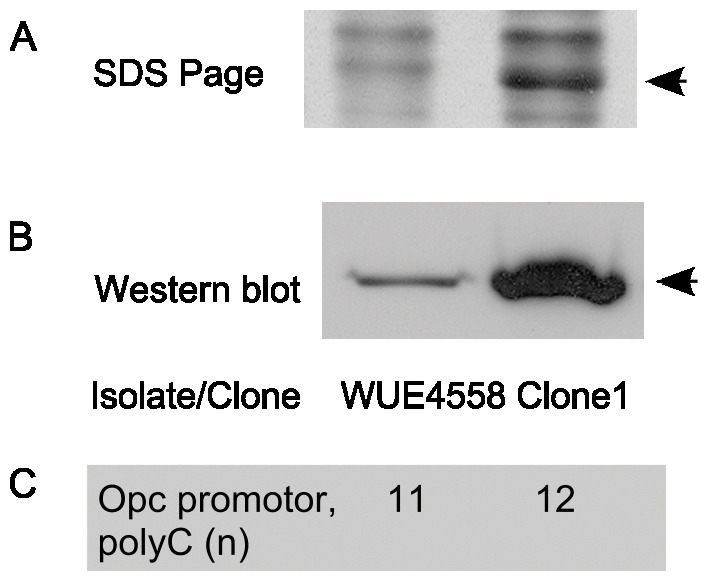
Mutant class I: increased expression of Opc by clones with enhanced serum resistance. (**A**) Coomassie blue stained SDS PAGE showing a prominent band at 26–28 kDa (arrow) in one of the resistant clones (clone 1). WUE4558 is the parental strain. (**B**) Detection of enhanced Opc-expression in clone 1 by Western blot developed with the Opc-specific monoclonal antibody B306. (**C**) The number of cytosine residues in the homopolymeric tract of the *opc* promoter is provided, which has been shown to dictate Opc expression [24].

Analysis of the whole cell lysate of clone 1 by SDS-PAGE revealed an increased expression of a 26–28 kDa protein (Fig. 2A). Mass spectrometry of the excised band showed that Opc was a major component of the band (peptide/protein coverage of 43%, 13 matching sequences and an exponentially modified protein abundance index of 2.72). Increased expression of the *opc* mRNA and of the Opc protein were confirmed by reverse transcription PCR, flow cytometry (data not shown) and Western blot analysis with the Opc specific monoclonal antibody B306, respectively (Fig. 2B). Of the 35 clones with enhanced serum resistance, as many as 31 showed increased expression of Opc as determined by Western blot analysis. The parental strain WUE4558 harbored eleven cytosine residues in the phase variable poly-C stretch of the *opc*-promoter, whereas the Opc high expressing variants harbored twelve (Fig. 2 C) as described by Sarkari et al. [24].

**Figure 3:**
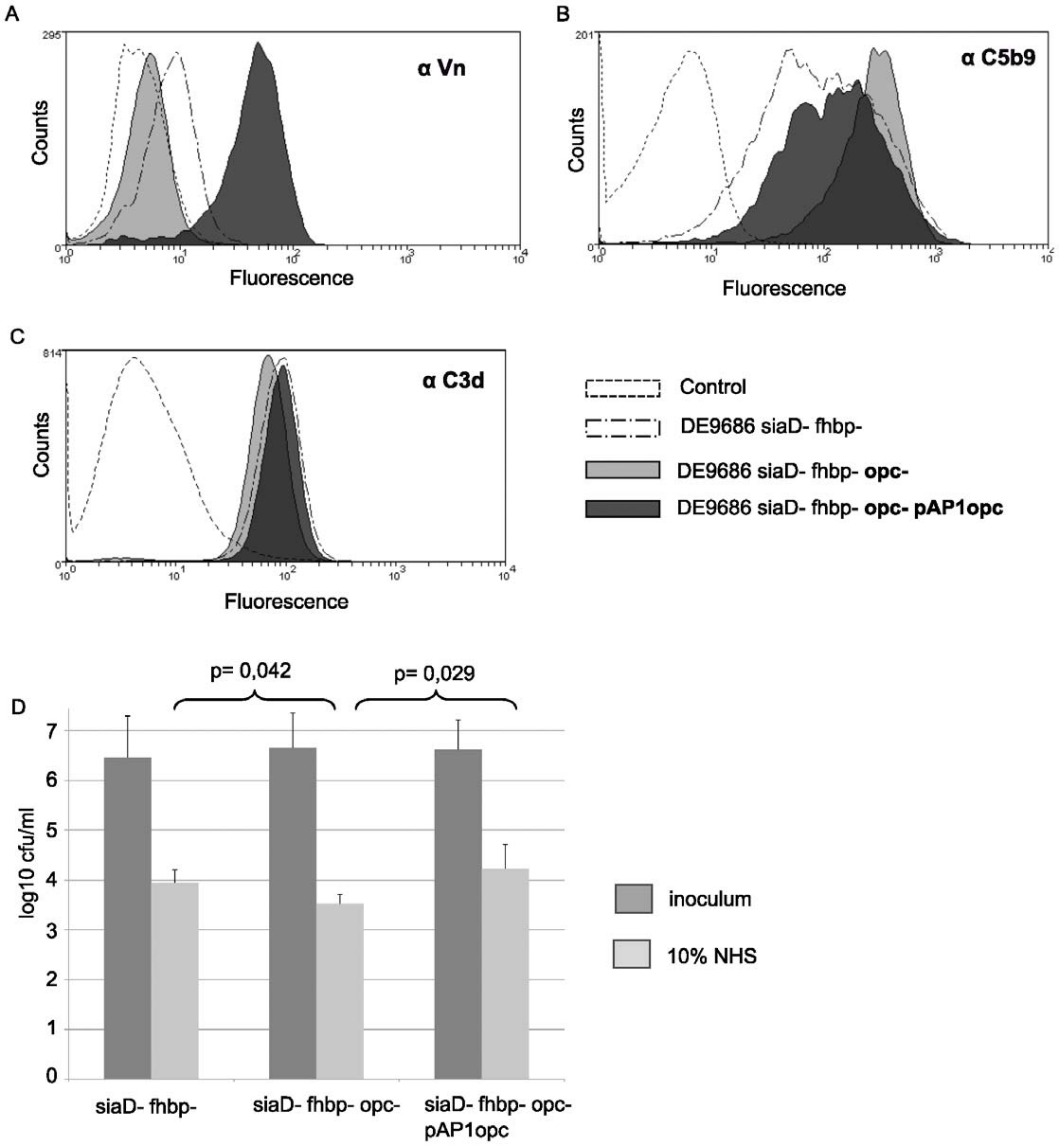
Analysis of complement attack by flow cytometry of isogenic derivatives of DE9686 *siaD*- *fhbp*-. (**A**) Enhanced vitronectin binding in the complemented mutant overexpressing Opc (DE9686 *siaD*- *fhbp*- *opc*- pAP1*opc*). (**B**) Membrane attack complex deposition on the Opc-positive strains DE9686 *siaD*- *fhbp*- and DE9686 *siaD*- *fhbp*- *opc*- pAP1*opc* in comparison to that on the *opc* knock out strain. (**C**) C3d load in DE9686 *siaD-, fhbp*- and the corresponding *opc* knock and *opc* complemented strain (**D**) Resistance towards 10% NHS of DE9686 *siaD*- *fhbp*-, DE9686 *siaD*- *fhbp*- *opc*-, and DE9686 *siaD*- *fhbp*- *opc*- pAP1*opc*. Data were compared by paired student's t-test.

Opc is an adhesin that was reported to bind vitronectin [9]. Loss of vitronectin binding was therefore confirmed in an *opc* knock-out strain (DE9686 *siaD*- *fhbp*- *opc*-); vitronectin binding was regained in its complemented derivative expressing Opc in trans (DE9686 *siaD*- *fhbp*- *opc*- pAP1*opc*) (Fig. 3A). Opc mutation had an impact on membrane attack complex (MAC) deposition (Fig. 3B), which was consistent with the previous hypothesis that recruitment of vitronectin reduces lethal attack by MAC [11], [25]. Opc mutation or complementation, however, did not affect complement factor C3 deposition as determined by flow cytometry (Fig. 3C). In line with the reduction of MAC deposition by enhanced Opc expression, moderate, but reproducible effects on survival in the serum killing assay were observed (Fig. 3D). This effect was only detectable in an *fhbp*-negative background, but not in the *fhbp*+-background (data not shown). The effect was in the range of what we saw for *fhbp* mutation. When we tested the *fhbp* mutation by comparing DE9686 *siaD*- with DE9686 *siaD*- *fhbp*-, the strain expressing fHbp was killed by 10^1,9^-fold, whereas additional knock-out of the *fhbp* gene resulted in a 10^2,9^-fold kill (n = 3, 10% NHS for 30 min, paired student's t-test: p = 0,008).

The predominance of serum resistant clones with enhanced Opc expression was the result of phase variation in the opc-promoter. We speculated that this was caused by the *mutS* mutation. However, seven of eleven clones identified in the screen of strain WUE4300 without *mutS* mutation (see above) also displayed enhanced Opc expression. This is an argument against a qualitative effect of *mutS* mutation in the screening assay.

### Mutant class II: LPS modification

**Figure 4:**
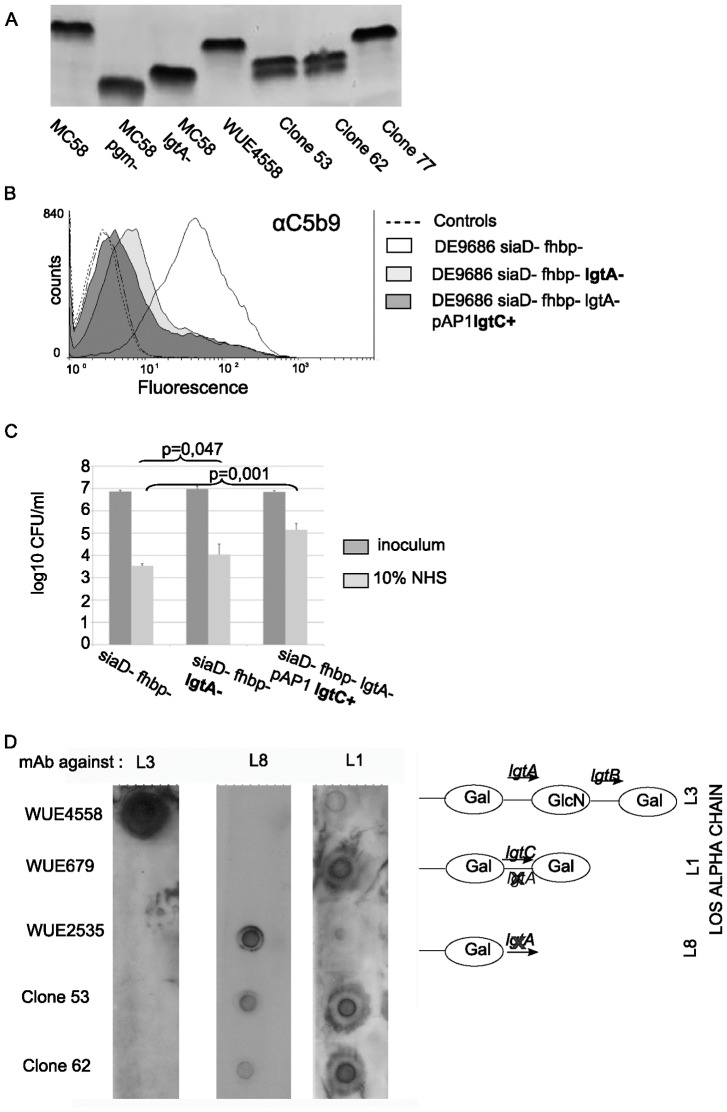
Mutant class II: demonstration of LPS immunotype changes and their consequences with regards to serum resistance. (A) Tricine gel electrophoresis revealed a double banding pattern for clone 53 and clone 62. As controls, strain MC58 (immunotype L3) and its *pgm* and *lgtA* (immunotype L8) mutants are shown, which have a truncation of four and two sugar residues, respectively. Clone 77 is shown as a further control, whose increased serum resistance is associated to pilin conversion, but not immunotype switches. (B) Flow cytometry analysis showing reduced deposition of membrane attack complex upon serum stress in strains DE9686 *siaD*- *fhbp*- *lgtA*- and DE9686 *siaD*- *fhbp*- l*gtA*- pAP1*lgtC_#53_* in comparison to strain DE9686 *siaD*-*fhbp*-. (C) Resistance towards 10% NHS was significantly enhanced in DE9686 *siaD*- *fhbp*- l*gtA*- and DE9686 *siaD*- *fhbp*- *lgtA*- pAP1*lgtC_#53_*, when compared to DE9686 *siaD*- *fhbp*-. Data were compared by paired student's t-test. (D) Immuno dot blots demonstrating the immunotypes of clones 53 and 62 both expressing L1 and L8 LPS. The structure of the terminal sugar residues of meningococcal LPS is schematically depicted for the three type strains (WUE4558, L3; WUE679, L1 type strain; WUE2535, lgtA knock out strain, L8; for further information see Table 1).

There were four clones with increased serum resistance, which did not show enhanced Opc expression. Two of those clones, i.e. clones 53 and 62, upon LPS analysis by tricine gel electrophoresis revealed smaller LPS species than in the parental strain and a double banding pattern suggesting two LPS species of different molecular weight (Fig. 4A). Because the molecular weight of one LPS band resembled that of an *lgtA* mutant, whose terminal lacto-N-neotetraose is truncated by two sugar residues, the phase variable homopolymeric tract of the *lgtA* gene was sequenced [26]. The length of the tract was eight guanine residues in the parental strain, whereas it was nine in the mutants, leading to inactivation of *lgtA*. Accordingly, the LPS of clones 53 and 62 reacted with an L8 antibody in contrast to the parental strain, which reacted with an anti L3,7,9 antibody in LPS dot-blot (Fig. 4D) and ELISA (data not shown). We speculated that the double banding pattern was associated with phase-variation of a second gene, i.e. *lgtC*, which phase-variably adds a galactose to the galactose of the truncated lacto-N-neotetraose, changing the immunotype of the LPS molecule from L8 to L1 [26]. The immunotype L1 was confirmed in clones 53 and 62 by dot blot analysis (Fig. 4D). Sequencing of the intragenic polyG tract of the *lgtC* gene revealed 12 G in the parental strain, and 13 G in clones 53 and 62, leading to expression of the galactosyltransferase encoded by *lgtC*. The results suggest that phase variation of the *lgtA* and *lgtC* genes altered the LPS immunotype to a mixture of L8 and L1, which in the *siaD*- *fhbp*- *lst*- background of the screening assay increased serum resistance.

To confirm the hypothesis that L8 and L1 support survival of ST-41/44 cc meningococci in NHS, an isogenic mutant of strain DE9686 *siaD*- *fhbp*- was constructed with an *lgtA* deletion (immunotype L8). This mutant was complemented in trans with *lgtC* of clone 53 to yield the genotype DE9686 *siaD*- *fhbp*- *lgtA*- pAP1*lgtC*_#53_ and the LPS immunotype L1. The mutants in comparison to DE9686 *siaD*- *fhbp*- showed a reduced deposition of MAC in flow cytometry (Fig. 4B). Furthermore, there was more than 10 fold increase of survival in the serum killing assay when the parental strain (L3,7,9) and the L1 expressing mutant were compared (Fig. 4C). This finding supported the assumption that LPS variation provided a selective advantage in the screening assay for the class II mutants.

**Figure 5:**
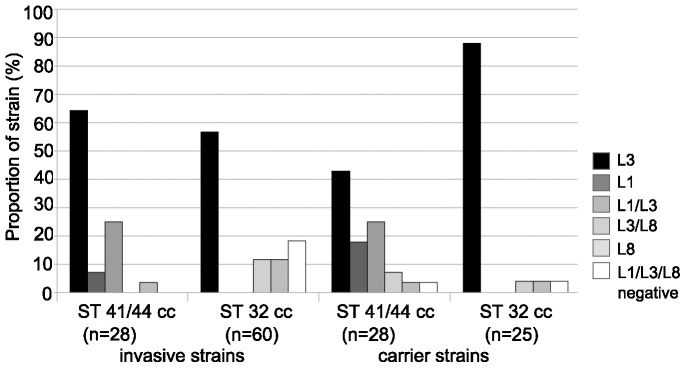
Presence of LPS immunotypes L1, L3 and L8 in ST-41/44 cc and ST-32 cc meningococci. Meningococcal LPS was analyzed by immuno dot blots. The isolates were obtained from invasive disease (German reference laboratory for meningococci) and healthy carriers [28], respectively.

We demonstrated that an immunotype switch contributes to serum resistance in vitro. We therefore asked whether and L8 and L1 are prevalent in strains of the ST-44/41 cc from disease and carriage. ST-32 cc strains were used as a control, because these strains do not express immunotype L1 due to a lack of *lgtC* [27]. The strains were obtained from the German reference laboratory for meningococci and the Bavarian meningococcal carriage study [28], respectively. In contrast to ST-32 cc strains, the L1 immunotype was often observed in ST41/44 complex strains, irrespective of whether the strains were isolated from carriage or invasive disease (Fig. 5). This finding suggests that LPS immunotype switching to L1 in the ST-41/44 cc is compatible both with carrier state and the propensity to cause invasive disease.

### Class III mutants: variation in PilE

**Figure 6:**
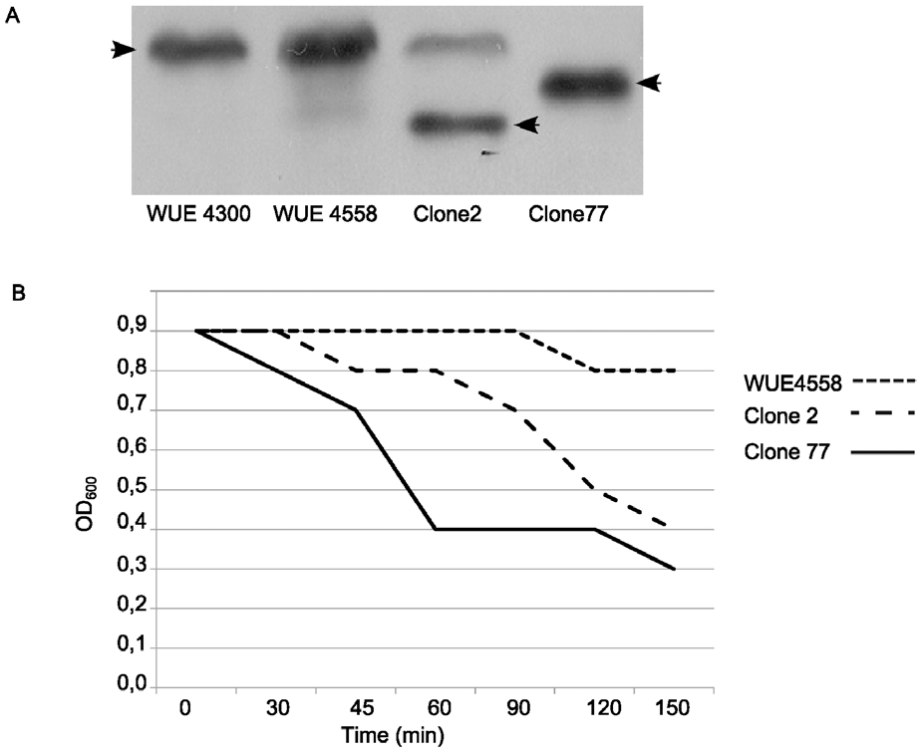
Mutant class III: antigenic variation of pilin and its impact on autoaggregation. (**A**) Immunoblot demonstrating class II pilin by antibody SM1(arrows). Both clones 2 and 77 revealed altered migration of PilE. (B) Autoaggregation assay: both clones 2 and 77 show accelerated rates of autoaggregation. Bacterial suspensions were kept without agitation for 150 min and the optical density was measured repeatedly to assess clearance of the suspension by autoaggregation and sedimentation.

Clones 2 and 77 neither showed enhanced Opc expression, nor displayed altered LPS immunotypes (data not shown). SDS-PAGE analysis revealed that both clones lacked a 20 kDa band in comparison to the parental strain. Western blot with the class II pilus specific antibody SM1 [29], revealed an altered pilin (PilE) size in clones 2 and 77 (Figure 6A). While clone 2 obviously harbored a mixed pilin population comprising a pilin of the size of parental strain and a fraction with faster migration in the SDS PAGE, clone 77 appeared to be homogenous with a single band migrating at elevated velocity compared to WUE4558. Both clones 2 and 77 showed enhanced serum resistance upon re-test in the serum killing assay with 10% and 20% NHS (Fig. 1C).

Pilin variation has been implicated in changes of autoaggregation. We therefore investigated autoaggregation by measurement of the reduction of the optical density of non-agitated liquid cultures. Both clones 2 and 77 showed enhanced autoaggregation, which was most obvious for clone 77 (Fig. 6B).

**Figure 7:**
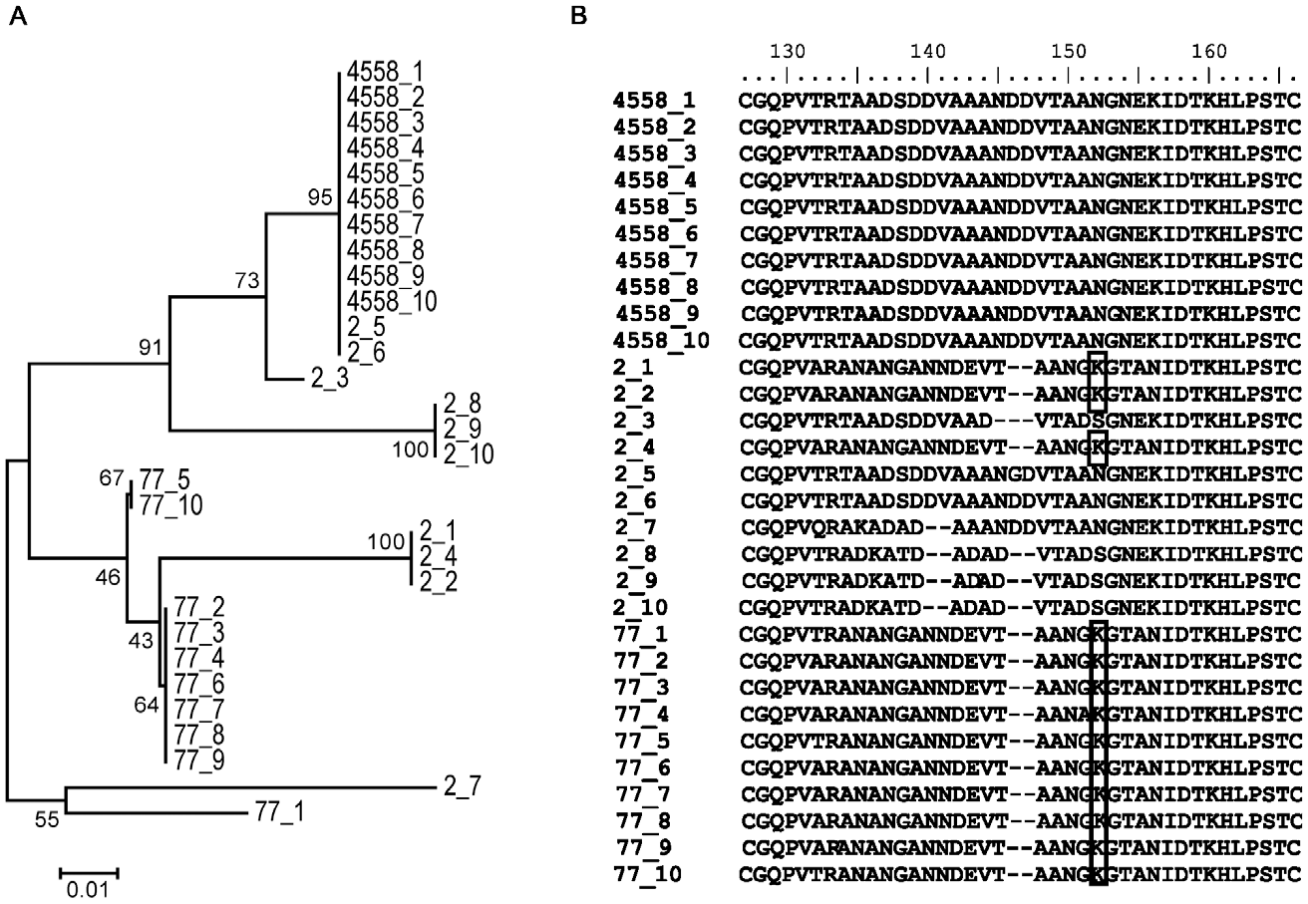
Sequence analysis of pilin conversion events. (A) Neighbor joining tree of Pilin amino acid sequences of individual colonies picked from WUE4558 and clones 2 and 77. The tree was computed with MEGA5 (http://www.megasoftware.net/; p-distance method; 168 positions of the aligned dataset). A bootstrap test was applied (2000 replicates). (B) Deduced amino acid sequences of the hypervariable region D flanked by two cysteine residues [32]. A lysine residue previously associated to pilus bundle formation [32] is highlighted by boxes.

To identify the reason for the SM1 banding pattern, we sequenced the *pilE* gene of the parental strain and the mutants, since silent *pilS* loci might replace the *pilE* of the parental strain by intrachromosomal recombination, a process designated as pilin conversion [30], [31]. Ten individual colonies were picked after plating and analyzed for both mutants and for the parental strain. To visualize the diversity of the amino acid sequences, a neighbor joining tree was constructed confirming that sequences derived from clone 2 were fairly diverse, which was in line with the observation of two distinct bands in the pilin Western blot (Fig. 6A). In contrast, clone 77 was more uniform and clearly distinct from the parental strain WUE4558. The PilE sequences (Fig. 7B) were highly diverse within the hypervariable region (D region) flanked by two cysteine residues, which has been described previously [32]. All colonies of the clone 77, 3 of 10 colonies of clone 2, but none of the colonies derived from WUE4558 harbored a particular lysine residue within the hypervariable region D. This lysine residue was previously described to be associated with increase bundling of pili [32].

**Figure 8:**
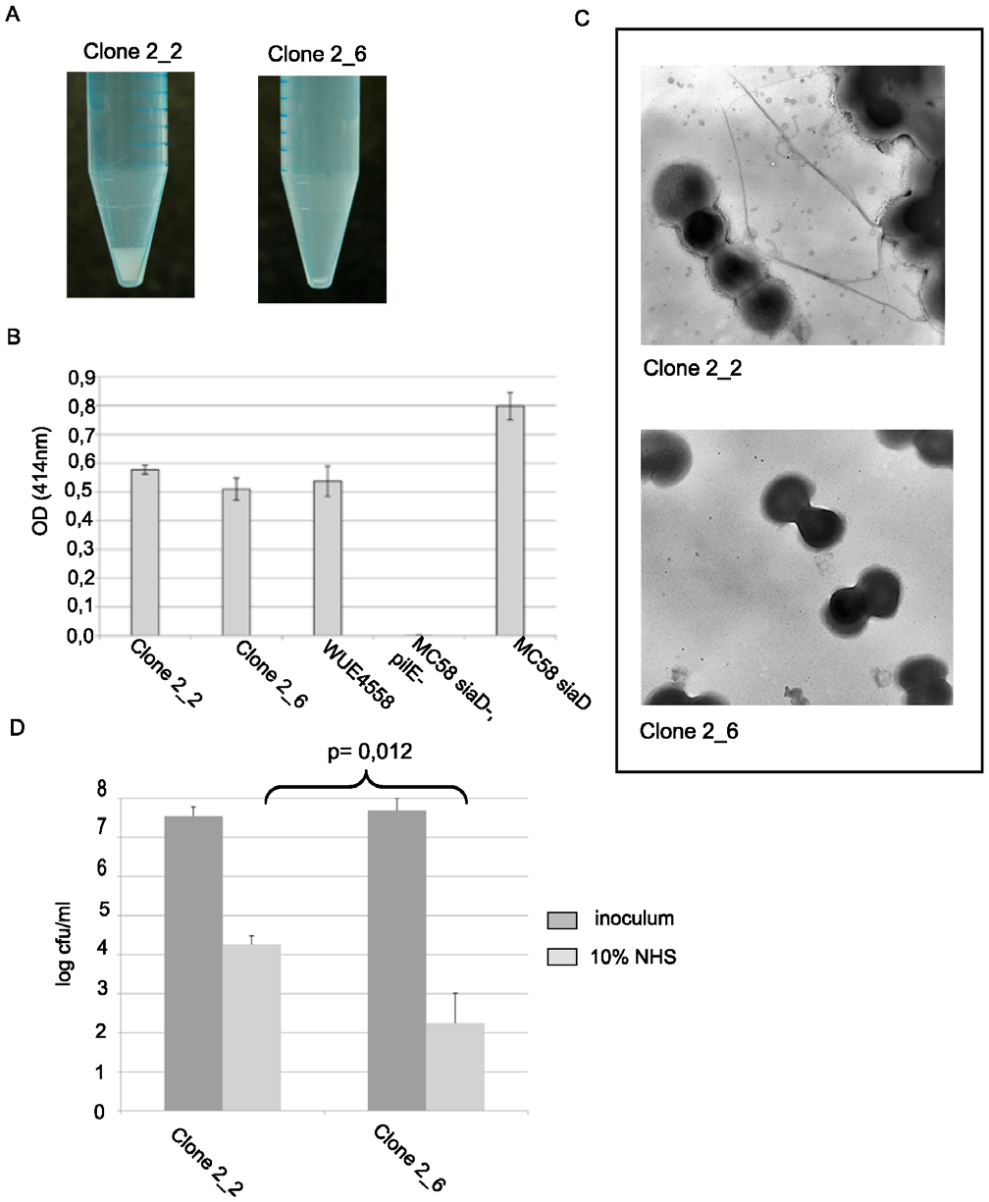
Comparison of clones 2_2 and 2_6. (A) Photograph showing autoaggregation of bacterial suspensions after 90 min. (B) Analysis of surface exposed pilin by ELISA. Clones 2_2 and 2_6 were compared with the parental strain WUE4558, the strain WUE3854 (pilE knock-out, negative control), and WUE3240 (MC58 siaD-, positive control). (C) Transmission electron micrographs of meningococci and pilus bundles obtained with a magnification of 16,000-fold. Pilus bundles were observed with clone 2_2, but not with clone 2_6. (D) Serum killing assay employing 10% normal human serum (NHS) on clones 2_2 and 2_6. Data were compared by a paired student's t-test.

Two derivatives of clone 2, i.e. clones 2_2 and 2_6, were obtained by colony picking from plate culture of clone 2 and were consecutively analyzed further. Clone 2_2 harbored a lysine previously associated with bundling of pili, clone 2_6 did not (Fig. 7B). Furthermore, increased autoaggregation of clone 2_2 was confirmed by comparison to 2_6 (Fig. 8A). This was not due to quantitative differences in surface piliation, as shown by ELISA employing the anti PilE antibody SM1 (Fig. 8B). Electron microscopy showed that pilus bundles were only observed in clone 2_2, which was in line with increased autoaggregation (Fig. 8C). To verify whether PilE bundle formation and autoaggregation differences in these closely related clones were associated with increased resistance towards human serum, a serum killing assay was conducted. Clone 2_2 turned out to be more resistant than 2_6 (Fig. 8D).

None of the mutations described above affected serine residues reported to be targets for glycosylation or addition of phospho-glycerol or phosphorylcholine. Nevertheless, DNA sequencing revealed that the increased migration velocity of the pilin variants most likely is not due to differences in the number of amino acids, but rather suggests that posttranslational modification may play a role. Therefore, the phase variable genes *pglA*, *pglB2*, *pglE*, *pglH*, and *pptA*, which are involved in post-translational modification, were analyzed by DNA sequencing for the presence of size variation of polymeric tracts. This analysis did not trace any differences between the parental stain and the mutant strains [33], [34]. *pglA* and *pglE* were both out of frame and thus presumably not active as has been suggested for ST-41/44 cc strains [35]. Thus, there was no molecular evidence for differences in the activity of enzymes required for posttranslational modification of pili.

The smaller size of PilE of the mutants was reminiscent of secreted pilin described previously [36]. By cleavage of an N-terminal fraction of the pilus subunit PilE, pilin maybe secreted into the environment. We therefore compared whole cell lysates and precipitated proteins from culture supernatants by Western blot analysis to identify increased pilin secretion in the mutants in comparison to the parental strain. The analysis did not reveal evidence for increased pilin secretion (data not shown).

In conclusion, class III mutants represented pilin variation caused by pilin conversion with effects on autoaggregation most likely associated to the presence of a lysine residue in region D. We postulate that enhanced serum resistance was caused by increased clumping of bacteria providing physical protection from complement attack.

## Discussion

In 2003, Geoffroy et al. analyzed a genome wide meningococcal transposon insertion library for genes essential for resistance against complement attack [12]. The work was pioneering as it was the starting point of a comprehensive resource of mutants of meningococcal genes. Nevertheless, almost exclusively mutations in capsule and LPS biosynthesis genes were found to render the strains serum susceptible. While on the one hand this finding demonstrates the extraordinary importance of these structures for serum resistance, it underlines on the other the necessity to work with mutants lacking dominant complement defense mechanisms, if other contributors to serum resistance are being aimed at. In this study, the latter was achieved by using an unencapsulated strain lacking fHbp and LPS sialylation. Furthermore, an additional *mutS* knock-out increased mutation rates and phase variation rates. Unexpectedly, the *mutS* mutation neither increased the number of serum resistant clones, nor the proportion of Opc overexpressing variants in comparison to a *mutS* expressing strain. This is in contrast to the finding of Bayliss et al. who could demonstrate that escape from the lethal action of a bactericidal antibody directed against an LPS epitope was enhanced by accelerated phase variation due to *mutS* mutation [37]. Therefore, there is currently no unambiguous evidence from in vitro experiments that serum resistance is supported by hypermutation. Nevertheless, hypermutating variants have been described for epidemic serogroup A disease [38]. This epidemiological finding may relate to a role of hypermutation in transmission and rapid adaptation to new niches rather than to resistance to human serum.

Three classes of mutants were enriched by serum treatment. Class I and class II mutants were the result of phase variation, either extragenic in the promoter (*opc*) or intragenic in LPS biosynthesis genes (*lgtA* and *lgtC*). Furthermore, intrachromosomal recombination was employed by the bacteria to achieve pilin variation. The predominant role on phase variation and intrachromosomal recombination is probably explained by the experimental set-up which selects for spontaneously evolving mutations in surface structures, but not for alterations of gene regulation in response to a lethal complement attack.

Dominant or well established complement resistance factors were eliminated from the test strain, i.e. the capsule, fHbp, and LPS sialylation. Not surprisingly, the individual contribution of each of the identified factors, when re-tested in isogenic mutant strains and their complemented counterparts, was overall small when compared to what is expected for the capsule [2]. Nevertheless, it was well in the range of what we observe for fHbp mutation alone - and of what is reported for fHbp [7]. This suggests that Opc expression, variation of LPS immunotype and pili are minor factors probably acting synergistically with established factors such as fHbp or which step in if fHbp or other factors are expressed at very low levels. Their specific contribution may depend on the clonal lineage. An example for this is the LPS immunotype L1, which can only be synthesized in ST-41/44 cc strains, but not in ST-32 cc strains, because the latter lack the *lgtC* gene [39]. Also the Opc protein is not present in all meningococcal lineages, as the ST-11 cc causing a large proportion of serogroup C disease, does not host the gene [40].

### Class I mutants

The vast majority of mutants expressed more Opc than the parental strain. The Opc protein is a surface exposed immunogenic adhesin of *N. meningitidis* present in an experimental outer membrane vesicle vaccine used for vaccination against serogroup B disease [41]. Its use in vaccination is hampered by the fact that not all strains harbor the gene and that its expression is phase variable [24]. Opc binds vitronectin and fibronectin to interact with host cells and tissue structures [9]. Furthermore, vitronectin binding by Opc is interesting in the context of serum resistance, because vitronectin many years ago has already been shown to be an inhibitor of the terminal complement complex [42]. Opc binding of vitronectin was reminiscent of observations in *Moraxella catarrhalis*, where the surface protein UspA2 binds vitronectin, which contacts the late complement component C9 which in turn inhibited membrane attack complex associated killing [43]. Griffiths et al. recently demonstrated that Msf, but also Opc bind vitronectin to inhibit the membrane attack complex [11]. Indeed, the detailed report by Griffiths et al. suggests co-operative action of Opc and Msf. Our report confirms the findings by Griffiths et al. in that Opc is a contributor to serum resistance. We demonstrate that in the context of capsule and fHbp deletion Opc over-expression is a strategy of survival in human serum.

### Class II mutants

The second class of mutants comprised LPS immunotype switches from L3,7,9 to L1 and L8 generated by phase variation. The parental strain like many other serogroup B meningococci expressed the L3 immunotype, with an outer core of terminal galactose, followed by N-acetyl glucosamine and galactose. The L8 immunotype is characterized by a truncated polysaccharide chain with an outer core consisting only of galactose. It is the result of phase variation in *lgtA* encoding a glycosyltransferase [26]. The L1 immunotype can only evolve from the L8 immunotype. A terminal galactose is added by a transferase encoded by the phase variable *lgtC* [27]. Among the major meningococcus B lineages, the galactosyltransferase LgtC is found in strains of the ST-41/44 cc, but not in the ST-32 cc [27]. As a consequence, L1 was not observed in ST-32 cc strains. It is important to note that immunotype L1 strains are not susceptible to the bactericidal activity of antibodies directed against L3 [44]. Under the experimental conditions applied herein, serum resistant clones with L8 and L1 LPS might therefore have been selected due to the presence of serum antibodies against L3 LPS. Within the frame of this study we were, however, unable to prove this hypothesis. Several experimental approaches to identify binding of antibodies to LPS, i.e. by ELISA, Western blot, and immuno dot-blot failed to detect specific signals, when L3 LPS preparations were compared with L8 LPS. Future studies should utilize more sensitive strategies and defined oligosaccharides.

The LPS switch was associated with spontaneous phase variation of *lgtA* and *lgtC*. Isogenic *lgtA* mutation and complementation with in frame *lgtC* revealed that both the L8 and the L1 immunotypes were beneficial to the bacteria under serum stress.

Sialylation of LPS is associated with higher serum resistance in gonococci, which bind fH via sialylated lacto-N-neotetraose [45]. L1 LPS can be sialylated in the gonococcus [46]. Nevertheless, sialylation explicitly did not play a role in our system, because the LPS sialyltransferase gene was inactivated.

In a cross-sectional study from the 1990s, L1 was more frequently found among strains isolated from healthy carriers than from invasive disease [47]. However, the strains were not controlled for clonal lineage. We therefore stratified our analysis to ST-41/44 cc and ST-32 cc meningococci; by this approach, we could not reproduce the published findings [47]. L3 dominated in both ST-32 cc and ST-41/44 cc. Nevertheless, considerable numbers of L1 strains were found in ST-41/44 cc strains from invasive disease and carriage. Therefore, L1 and L8 cannot be considered as typical carriage LPS immunotypes, but also appear in a significant fraction of disease isolates. The results of the previous study might have been influenced by carriage associated clonal lineages, which do not occur in invasive disease.

### Class III mutants

We identified two clones altered in pilin with regard to size. The modification was the result of *pilE* switches, which in the Neisseria are mediated by intrachromosomal recombination of silent *pilS* loci into the *pilE* gene. Analysis of several colonies derived from one clone revealed some degree of additional variation reminiscent of the finding that isolates from a single patient differ in the expressed PilE [48]. The *pilE* mutation frequency might have been enhanced in our experimental setting due to *mutS* mutation as recently shown for gonococci [49]. The mutation was found in a hypervariable C-terminal region D of the pilus first described for gonococci [50], which is located between the cys1 and cys2 domains [32]. The hypervariable region, where the majority of polymorphisms were found also in this study, is located on the surface of the pilus structure [51], [52].

Until now, there is no clear published evidence that type IV pili contribute to serum resistance. The only information comes from work on the interaction of the complement regulator C4 binding protein (C4BP), which appears to bind to gonococcal PilC, a pilus associated protein [53]. Enhanced binding of C4BP might regulate the classical pathway of complement activation. However, preliminary evidence from our laboratory makes an involvement of PilC unlikely, as we were not able to demonstrate an association of PilC expression and the presence of pilin mutations specific to clone 77. There has been evidence that reduction of pilus glycosylation is associated with reduced serum resistance, however this effect was largely attributed to a concomitant reduction of capsule polysaccharide expression [54].

The PilE alterations were accompanied by an increased autoaggregation. Chamot-Rooke et al. demonstrated that phosphoglycerol in meningococci is inhibiting bacterial aggregation by increased expression of *pptB* upon contact to cells and consecutive bacterial proliferation [55]. Another minor pilin associated with aggregation is PilX [56]. However, using a polyclonal antibody [57] we were not able to identify any differences in PilX expression (data not shown). We furthermore did not identify any changes in phase variable genes involved in posttranslational modification of pili. In addition, there were no consistent mutations of serine residues acting as target sites for modification.

Analysis of the peptide sequences revealed that all colonies picked from a culture of the hyperaggregating clone 77 harbored a lysine in the C terminal hypervariable region upstream of the cys2 domain, which was described by Marceau et al. to contribute to bundle formation of pili [32]. This lysine residue was absent in all of the hypoaggregative derivatives of WUE4558. The replacement of the hypervariable region of WUE4558 was therefore associated with increased aggregation, and increased aggregation can partially be explained by published evidence regarding the role of a single lysine residue in the C-terminal hypervariable region, which is contributing to the pilus bundle's outer surface [51].

It is plausible that increased autoaggregation reduces the attack area of serum complement. There has been evidence from *Vibrio cholerae*, which also expresses a type IV pilus, that certain mutations in the N-terminus, which abrogate autoaggregation, but not piliation, enhance killing by complement [58]. The interpretation was that aggregates physically hinder complement to interact with the bacterial cells. However, the interpretation was limited by the finding that not all mutations resulting in reduced autoaggregation also rendered the bacteria serum susceptible. It maybe that size of aggregates plays a role. Alternatively, other yet unidentified interactions of pili with the complement system need to be addressed. *Haemophilus* phosphorylcholine decoration of LPS reduced serum resistance [59] as a result of CRP binding to phosphorylcholine and consecutive activation of the classical pathway of complement [60]. Interestingly, CRP can also bind to neisserial pili modified with phosphorylcholine [61]. However, our initial attempts to demonstrate increased CRP binding to the mutants selected in this study failed, and the analysis of phosphorylcholine on pilus preparations of the clones was inconsistent (data not shown). Future studies therefore need to establish a functional relation between lack of phosphorylcholine decoration of pili and complement activation. Also the above mentioned C4BP binding to PilC needs to be studied in detail, because PilE variation might concur with PilC variation [48].

In conclusion, the screening approach applied in this study provided data that contribute to the understanding of serum resistance in meningococci and the importance of intragenomic variation for survival in this hostile environment. These bacteria employ a redundancy of factors to flexibly adapt to complement attack. Our future studies will concentrate on the role of pilus variation for meningococcal serum resistance.

## Materials and Methods

### Strains and mutants

All meningococcal isolates and mutant strains are listed in Table 1. Strain DE9686 is an isolate from the cerebrospinal fluid of a 4 year-old patient from the county Aachen in Germany [21]. It was typed by the German reference laboratory for meningococci as B:P1.7-2,4:F1-5:ST-42:ST-41/44 cc, where P1 is PorA with its variable regions 1 and 2, F is FetA with a single variable region, ST is multilocus sequence type, and cc is clonal complex. The capsule polysaccharide synthesis gene *siaD* (syn.: *synD*), the lipopolysaccharide sialyltransferase gene *lst*, and the *fhbp* gene were deleted as described [2], [62], [63]. The resulting organism WUE4300 was resistant to chloramphenicol, kanamycin, and spectinomycin, respectively. To enhance the strain's mutation rate, the *mutS* gene was knocked out by inserting an erythromycin resistant gene into the unique NdeI site as follows: Primers AB9 (5′CGCCGTTTCCCCAATGATG-3′) and AB10 (5′-AGGCCGTCGAAAGTGGAAG-3′) were used to amplify *mutS* of strain MC58 (kindly provided by Richard Moxon, Oxford). The PCR product was cloned into pCR-XL-TOPO TA (life Technologies, Darmstadt, Germany) and the resulting plasmid was digested with NdeI and ligated with an erythromycin resistance gene. The resulting plasmid pAB7 was used for transformation of strain WUE4300. Correct recombination was confirmed by Southern blot analysis. The resulting organism was designated WUE4558, and was used throughout the project. Growth on solid media (GC Agar, BD) and in Mueller Hinton Broth (Difco) in 96 well round bottom plates (Sarstedt) took place at 37°C with 5% CO_2_. Functional characterization of genes was consecutively conducted using strain WUE4240, which is a DE9686 derivative with mutations in *siaD* and *fhbp*. WUE4240 was used because WUE4558 already harbored four resistance cassettes for selection and no other antibiotic resistance cassettes were available for additional mutations and complementations. Strain WUE4240 was used to mutate *opc* as described using a kanamycin resistance cassette resulting in strain WUE4712 [64]. Furthermore, *lgtA* was knocked-out in strainWUE4240 as described generating strain WUE4785 [65]. WUE4712 and WUE4785 were complemented in trans using the complete *opc* and *lgtC* genes, respectively, expressed from the plasmid pAP1 under the control of a *porA* promoter generating strains WUE4748 and WUE4786 [57]. Collections of genetically typed invasive and carrier isolates of the ST-41/44 and ST-32 cc were obtained from the German reference laboratory for meningococci and from the Bavarian carriage study [28].

**Table 1:** Strains used in this study.

Parental strain	Derivative	Sequence type (ST)	Clonal complex (cc)	Serogroup	Serogeno-group	Epidemio-logy	Year/Geography	Wild type (wt)/Genetically modified organism (GMO)	Plasmid content	Resistance profile**	Reference
DE9686		42	41/44	B	B	Disease	2004/DE	wt	−	−	[21]
DE9686	WUE4240	42	41/44	NSG	B	n.a.	n.a.	GMO (siaD-, fhbp-)	−	Cm, Spc	
DE9686	WUE4300	42	41/44	NSG	B	n.a.	n.a.	GMO (siaD-, lst-, fhbp-)	−	Cm, Kan, Spc	
DE9686	WUE4558	42	41/44	NSG	B	n.a.	n.a.	GMO (siaD-, lst-, fhbp-, mutS-)	−	Cm, Kan, Spc, Ery	
DE9686	WUE4712	42	41/44	NSG	B	n.a.	n.a.	GMO (siaD-, fhbp-, opcA-)	−	Cm, Spc, Kan	
DE9686	WUE4748	42	41/44	NSG	B	n.a.	n.a.	GMO (siaD-, fhbp-, opcA- pAPopcA)	+	Cm, Spc, Kan, Ery	
DE9686	WUE4785	42	41/44	NSG	B	n.a.	n.a.	GMO (siaD-, fhbp-, lgtA-)	−	Cm, Spc, Kan	
DE9686	WUE4786	42	41/44	NSG	B	n.a.	n.a.	GMO (siaD-, fhbp-, lgtA-,pAP lgtC+)	+	Cm, Spc, Kan, Ery	
126E	WUE679	n.d.	n.d.	C	n.d.	Disease	n.d.	wt	−	−	[69]
MC58		74	32	B	B	Disease	1985/UK	wt	−	−	[70]
MC58	WUE3240	74	32	NSG	B	n.a.	n.a.	GMO (siaD-)	−	Cm	[71]
MC58	WUE3854	74	32	NSG	B	n.a.	n.a.	GMO (siaD-, pilE-, gfp)	+	Cm, Kan, Ery	[57]
MC58	WUE2535	74	32	NSG	B	n.a.	n.a.	GMO (lgtA-)	−	Kan	[65]

** CM, chloramphenicol; Ery, erythromycin; Kana, kanamycin, Spc, spectinomycin, n.d., not determined; DE, Germany; UK, United Kingdom; n.a., not applicable; NSG, not serogroupable.

### Human serum and antibodies

One lot of pooled human complement serum (normal human serum, NHS) was purchased from Innovative Research inc. (USA) via Dunn Labortechnik (Germany) and used throughout the study with the exception of vitronectin detection experiments. Monoclonal antibody mAB B306 was used to detect OpcA and was a kind gift of M. Achtman [66]. Antibodies against the LPS immunotypes L1, L8, and L3,7,9 were a kind gift of W. Zollinger [67]. The pilus subunit PilE was detected with monoclonal antibody SM1, which was kindly provided by M. Virji [29].

### Determination of mutation and phase variation rates

Spontaneous mutation rates were measured as the proportion of rifampicin resistant colonies [16]. Strains WUE4300 (DE9686 *siaD*- *fhbp*- *lst*-) and WUE4558 (DE9686 *siaD*- *lst*- *fhbp*- *mutS*-) were grown on GC agar overnight. Bacterial suspensions with OD_600_ 1 in PBS were plated in serial tenfold dilutions on GC agar and on GC agar containing 3 µg/ml rifampicin. Means of four independent experiments are provided as the ratio of surviving log_10_ cfu/ml devided by the log_10_ cfu/ml of input cells. Phase variation in the homopolymeric tract of the *opc* promoter was analyzed as follows: single colonies from overnight cultures on GC agar of strains WUE4300 and WUE4588 were grown for another six hours at 37°C on GC agar. Suspensions of single colonies were produced in PBS with OD_600_ 0.1. The *opc* promoter region was amplified with primers HC595 (5′-GAAACCGGACGAACCTAGATTC-3′) and HC590 (5′-ATCAGAATTTTATGCCGACGC-3′) using the Phusion® High-Fidelity PCR Kit (Thermo Scientific) and the homopolymeric tract was sequenced with primer HC376 (5′ATTGTAGTCGGATATGGTAAC-3′).

### Colorimetric assay for determination of serum mediated killing modified from [15]

Strain WUE4558 was grown overnight on GC agar plates. Bacteria were resuspended in 5 ml of Mueller Hinton (MH) broth (DIFCO) to OD_600_ 0.2. After one hour of growth at 37°C the culture was adjusted to OD 0.2 with pre-warmed (37°C) MH broth and grown for an additional hour to ensure optimal fitness of the bacteria. Consecutively, 10^7^ cfu/ml were incubated for 30 minutes with 10% NHS (Innovative Research) in Hanks' balanced salt solution (HBSS++, Invitrogen) containing 0.1% BSA at 37°C and 700 rpm (Eppendorf Thermo mixer). By this procedure >99.9% of the bacteria were killed. Surviving bacteria were grown on GC agar overnight. Single colonies were isolated and grown for approximately 15 hours on GC agar plates with grid. Single clones were transferred with a tooth pick into 200 µl MH Broth in 96 well polystyrol plates with round bottom (Sarstedt) and sealed with a polyester tape (NUNC) for shaking on a plate vortexer for one minute. Tapes were replaced by a loose sterile plastic lid and bacteria were grown for two hours. OD reads at 595 nm confirmed approximately equal numbers of bacteria in each well and bacteria were diluted to 10^−2^ in PBS in a new plate. Ten µl of this dilution were added to 40 µl HBSS++/0.1% BSA in the control plate or to 20 µl HBSS++/0.1% BSA in the serum stress plate, to which subsequently 20 µl of chilled 25% NHS diluted with HBSS++/0.1% BSA were added resulting in a final serum concentration of 10% NHS. After shaking for one minute the plates were incubated at 37°C/5% CO_2_ for 30 minutes. All samples were diluted with 37°C warm 150 µl MH broth which contained 2% glucose and approx. 0.002% bromocresol blue. Visual control and OD read (406 nm) were performed after 25 to 29 hours and 48 hours with a vortex step conducted beforehand. Growth of bacteria was indicated by yellow color.

### Standard serum killing assay employing plating of serial dilutions

MH broth was inoculated with bacteria from an overnight culture on GC agar to OD 0.2 and grown for one hour, diluted to OD 0.2 with MH broth and grown for an additional hour. Consecutively, pelleted bacteria were resuspended in HBSS++/0.1% BSA and 10^7^ bacteria were incubated with NHS in a final volume of 1 ml for 30 minutes at 37°C in and 700 rpm. Serial dilutions of the surviving bacteria were plated on GC agar.

### Immuno-assays

After SDS PAGE using 10 or 15% polyacrylamide gels and Western blotting, unspecific binding sites on nitrocellulose were blocked for 30 minutes with 5% skim milk in 1×PBS with 0.1% Tween 20, first antibody incubation in PBS/1% skim milk/0.1% Tween20 took place at 4°C overnight. PBS/0.1% Tween was used as washing buffer. Peroxidase labeled secondary antibody directed against mouse IgG/IgM (Jackson Immunoresearch) was detected with ECL Western blotting substrate (Pierce). For LPS immuno dot blots, the lipopolysaccharide was purified by microextraction using proteinase K digestion as described [68]. One µl of this LPS preparation was dotted on nylon membrane, rinsed with 20% ethanol, dried and developed as described above. Strain 126E was used as L1 positive control [67]. Strain MC58 *lgtA*- was used as the L8 positive control.

### Flow cytometry

Bacteria were grown on chocolate agar overnight at 37°C and 5% CO_2_. Few colonies were resuspended in 1 ml veronal-buffered saline (VBS)/BSA (5 mM 5,5-diethyl-barbituric acid, 145 mM NaCl, 0.5 mM MgCl_2_, 0.15 mM CaCl_2_ plus 0.5% BSA) and adjusted to OD_600_ 0.2. Bacteria were pelleted by centrifugation and resuspended in VBS/BSA supplemented with pooled human serum at the appropriate concentration. Samples were incubated at 37°C and 200 rpm. Serum stress was stopped by adding 400 µl of cold HBSS^++^/BSA (1× HBSS^−^,1 mM MgCl_2_, 0.15 mM CaCl_2_, 1% BSA). After a single washing step with HBSS^++^/BSA, bacteria were incubated with 50 µl of monoclonal antibodies anti-C5b9 (Dako; clone aE11), anti Opc (MAb B306), or rabbit anti C3d α humanC3d (DAKO), diluted 1∶50 in washing buffer for 30 minutes at 37°C (700 rpm). After another washing step bacteria were resuspended in a 1∶200 dilution of Alexa Fluor® 488 goat anti-mouse IgG antibody or Alexa-Flour 488 chicken anti rabbit IgG (Invitrogen), respectively, in HBSS^++^/BSA and incubated for 30 minutes at 37°C (700 rpm). Following a washing step, bacteria were fixed in 1 ml 1% formaldehyde in phosphate buffered saline (PBS) for at least 1 hour at 4°C in the dark followed by a washing step and resuspension in 400 µl PBS. Consecutively, bacteria were analyzed using a FACSCalibur flow cytometer (Becton Dickinson, Heidelberg; Germany).

To detect vitronectin binding to bacteria by flow cytometry, bacteria were incubated for 30 min with 10% heat inactivated serum in VBS. Incubation in VBS alone served as a negative control. A mouse anti human vitronectin antibody purchased from Quidel diluted 1∶200 was used to detect the protein after washing steps.

### Mass spectrometry

After separation of proteins from whole cell lysates using SDS PAGE and visualization of proteins with Coomassie blue or FireSilver staining (Proteome Factory, Germany), bands were excised from gels. Protein identification by nanoLC-ESI-MS/MS analysis was performed by Proteome Factory.

### Aggregation assay

Strains were grown to OD_600_ 0.7 in Mueller-Hinton broth, centrifuged at 4000 rpm, and re-suspended in HBSS++/0.1% BSA to reach OD_600_ 0.9. Aggregation of bacteria was monitored in 15 ml plastic tubes by OD600 measurement every 15 min.

### PilE ELISA

Pili on the surface of bacteria was measured with a whole-cell ELISA adapted from Helaine et al. [56]. In short, Bacteria were grown on GC agar plates overnight, resuspended in MH Broth to OD 0,2, incubated at 37°C until OD 0,6 was reached, and finally diluted to OD 0,2 in PBS. 96 well plates were coated with serial two fold dilutions of this suspension and dried without covers overnight at room temperature under a laminar airflow. Plates were washed five times with PBS-0.1% Tween. Bacteria were then incubated with 50 µl MAb SM1 directed against PilE diluted 1∶1000 in PBS-0.1% Tween-1% BSA-1% skimmed milk for 1 h at 37°C, washed five time with PBS-0.1% Tween following incubation with 50 µl Peroxidase anti mouse IgG/IgM diluted 1: 2500 in PBS-0.1% Tween for 1 hour at room temperature. After washing five times with PBS-0.1% Tween, peroxidase signal was developed with a solution of ABTS (Roche) prepared following the manufacturer's recommendations. Measurement in ELISA plate reader was done at 414 nm after 10 minutes and 20 minutes.

### Transmission Electron microscopy

10 µl of bacterial aggregates were taken from the tube bottom of the aggregation assay (see above) after 60 minutes of initiation of the assay. The bacterial suspensions were adsorbed to Formvar Carbon coated copper grids for 5 minutes. Grids were incubated for 5 min with 1,25% glutaraldehyde in PBS washed two times with distilled water and stained for 1 min with 2% uranyl acetate. Electron microscopy was conducted at the Department for Electron Microscopy at the Biocenter of the University of Wuerzburg.

## References

[1] Schneider MC, Exley RM, Ram S, Sim RB, Tang CM (2007) Interactions between Neisseria meningitidis and the complement system. Trends Microbiol 15: 233–240. 17398100 10.1016/j.tim.2007.03.005

[2] Vogel U, Weinberger A, Frank R, Muller A, Kohl J, et al (1997) Complement factor C3 deposition and serum resistance in isogenic capsule and LOS sialic acid mutants of serogroup B Neisseria meningitidis. Infect Immun 65: 4022–4029. 9317002 10.1128/iai.65.10.4022-4029.1997 PMC175578

[3] Vogel U, Claus H, von Muller L, Bunjes D, Elias J, et al (2004) Bacteremia in an immunocompromised patient caused by a commensal Neisseria meningitidis strain harboring the capsule null locus (cnl). J Clin Microbiol 42: 2898–2901. 15243035 10.1128/JCM.42.7.2898-2901.2004 PMC446252

[4] Rappuoli R (2000) Reverse vaccinology. Curr Opin Microbiol 3: 445–450. 11050440 10.1016/s1369-5274(00)00119-3

[5] Masignani V, Comanducci M, Giuliani MM, Bambini S, du-Bobie J, et al (2003) Vaccination against Neisseria meningitidis using three variants of the lipoprotein GNA1870. J Exp Med 197: 789–799. 12642606 10.1084/jem.20021911 PMC2193853

[6] Serruto D, Spadafina T, Ciucchi L, Lewis LA, Ram S, et al (2010) Neisseria meningitidis GNA2132, a heparin-binding protein that induces protective immunity in humans. Proc Natl Acad Sci U S A 107: 3770–3775. 20133713 10.1073/pnas.0915162107 PMC2840514

[7] Madico G, Welsch JA, Lewis LA, McNaughton A, Perlman DH, et al (2006) The meningococcal vaccine candidate GNA1870 binds the complement regulatory protein factor H and enhances serum resistance. J Immunol 177: 501–510. 16785547 10.4049/jimmunol.177.1.501 PMC2248442

[8] Lewis LA, Ngampasutadol J, Wallace R, Reid JE, Vogel U, et al (2010) The meningococcal vaccine candidate neisserial surface protein A (NspA) binds to factor H and enhances meningococcal resistance to complement. PLoS Pathog 6: e1001027. 20686663 10.1371/journal.ppat.1001027 PMC2912398

[9] Sa E Cunha C, Griffiths NJ, Virji M (2010) Neisseria meningitidis Opc invasin binds to the sulphated tyrosines of activated vitronectin to attach to and invade human brain endothelial cells. PLoS Pathog 6: e1000911 10.1371/journal.ppat.1000911 [doi]. 20502634 10.1371/journal.ppat.1000911 PMC2873925

[10] Singh B, Jalalvand F, Morgelin M, Zipfel P, Blom AM, et al (2011) Haemophilus influenzae protein E recognizes the C-terminal domain of vitronectin and modulates the membrane attack complex. Mol Microbiol 81: 80–98. 21542857 10.1111/j.1365-2958.2011.07678.x

[11] Griffiths NJ, Hill DJ, Borodina E, Sessions RB, Devos NI, et al (2011) Meningococcal surface fibril (Msf) binds to activated vitronectin and inhibits the terminal complement pathway to increase serum resistance. Mol Microbiol 82: 1129–1149. 22050461 10.1111/j.1365-2958.2011.07876.x

[12] Geoffroy MC, Floquet S, Metais A, Nassif X, Pelicic V (2003) Large-scale analysis of the meningococcus genome by gene disruption: resistance to complement-mediated lysis. Genome Res 13: 391–398. 12618369 10.1101/gr.664303 PMC430250

[13] Sun YH, Bakshi S, Chalmers R, Tang CM (2000) Functional genomics of Neisseria meningitidis pathogenesis. Nat Med 6: 1269–1273 10.1038/81380 [doi]. 11062540 10.1038/81380

[14] Rodriguez T, Lastre M, Cedre B, Fajardo EM, del Campo J, et al (2003) Validation of colorimetric assay to detect complement-mediated antibody-dependent bactericidal activity against serogroups B and C Neisseria meningitidis. Biologicals 31: 209–212. 12935810 10.1016/s1045-1056(03)00060-5

[15] Rodriguez T, Lastre M, Cedre B, del Campo J, Bracho G, et al (2002) Standardization of Neisseria meningitidis serogroup B colorimetric serum bactericidal assay. Clin Diagn Lab Immunol 9: 109–114. 11777839 10.1128/CDLI.9.1.109-114.2002 PMC119898

[16] Richardson AR, Stojiljkovic I (2001) Mismatch repair and the regulation of phase variation in Neisseria meningitidis. Mol Microbiol 40: 645–655. 11359570 10.1046/j.1365-2958.2001.02408.x

[17] Saunders NJ, Jeffries AC, Peden JF, Hood DW, Tettelin H, et al (2000) Repeat-associated phase variable genes in the complete genome sequence of Neisseria meningitidis strain MC58. Mol Microbiol 37: 207–215. 10931317 10.1046/j.1365-2958.2000.02000.x

[18] Martin P, van de Ven V, Mouchel N, Jeffries AC, Hood DW, et al (2003) Experimentally revised repertoire of putative contingency loci in Neisseria meningitidis strain MC58: evidence for a novel mechanism of phase variation. Mol Microbiol 50: 245–257 3678 [pii]. 14507378 10.1046/j.1365-2958.2003.03678.x

[19] Caugant DA, Bol P, Hoiby EA, Zanen HC, Froholm LO (1990) Clones of serogroup B Neisseria meningitidis causing systemic disease in The Netherlands, 1958–1986. J Infect Dis 162: 867–874. 2119401 10.1093/infdis/162.4.867

[20] Baker MG, Martin DR, Kieft CE, Lennon D (2001) A 10-year serogroup B meningococcal disease epidemic in New Zealand: descriptive epidemiology, 1991–2000. J Paediatr Child Health 37: S13–S19. 11885731 10.1046/j.1440-1754.2001.00722.x

[21] Elias J, Schouls LM, van de Pol I, Keijzers WC, Martin DR, et al (2010) Vaccine preventability of meningococcal clone, Greater Aachen Region, Germany. Emerg Infect Dis 16: 465–472. 20202422 10.3201/eid1603.091102 PMC3322024

[22] Joseph B, Schwarz RF, Linke B, Blom J, Becker A, et al (2011) Virulence evolution of the human pathogen Neisseria meningitidis by recombination in the core and accessory genome. PLoS ONE 6: e18441. 21541312 10.1371/journal.pone.0018441 PMC3082526

[23] Oster P, Lennon D, O'Hallahan J, Mulholland K, Reid S, et al (2005) MeNZB: a safe and highly immunogenic tailor-made vaccine against the New Zealand Neisseria meningitidis serogroup B disease epidemic strain. Vaccine 23: 2191–2196. 15755593 10.1016/j.vaccine.2005.01.063

[24] Sarkari J, Pandit N, Moxon ER, Achtman M (1994) Variable expression of the Opc outer membrane protein in Neisseria meningitidis is caused by size variation of a promoter containing poly-cytidine. Mol Microbiol 13: 207–217. 7984102 10.1111/j.1365-2958.1994.tb00416.x

[25] Hallstrom T, Trajkovska E, Forsgren A, Riesbeck K (2006) Haemophilus influenzae surface fibrils contribute to serum resistance by interacting with vitronectin. J Immunol 177: 430–436. 16785539 10.4049/jimmunol.177.1.430

[26] Jennings MP, Srikhanta YN, Moxon ER, Kramer M, Poolman JT, et al (1999) The genetic basis of the phase variation repertoire of lipopolysaccharide immunotypes in Neisseria meningitidis. Microbiology 145 (Pt 11) 3013–3021. 10589709 10.1099/00221287-145-11-3013

[27] Berrington AW, Tan YC, Srikhanta Y, Kuipers B, van der Ley P, et al (2002) Phase variation in meningococcal lipooligosaccharide biosynthesis genes. FEMS Immunol Med Microbiol 34: 267–275. 12443826 10.1111/j.1574-695X.2002.tb00633.x

[28] Claus H, Maiden MC, Wilson DJ, McCarthy ND, Jolley KA, et al (2005) Genetic analysis of meningococci carried by children and young adults. J Infect Dis 191: 1263–1271. 15776372 10.1086/428590

[29] Virji M, Heckels JE, Potts WJ, Hart CA, Saunders JR (1989) Identification of epitopes recognized by monoclonal antibodies SM1 and SM2 which react with all pili of Neisseria gonorrhoeae but which differentiate between two structural classes of pili expressed by Neisseria meningitidis and the distribution of their encoding sequences in the genomes of Neisseria spp. J Gen Microbiol 135: 3239–3251. 2483993 10.1099/00221287-135-12-3239

[30] Meyer TF, Billyard E, Haas R, Storzbach S, So M (1984) Pilus genes of Neisseria gonorrheae: chromosomal organization and DNA sequence. Proc Natl Acad Sci U S A 81: 6110–6114. 6148752 10.1073/pnas.81.19.6110 PMC391869

[31] Swanson J, Bergstrom S, Robbins K, Barrera O, Corwin D, et al (1986) Gene conversion involving the pilin structural gene correlates with pilus+ in equilibrium with pilus- changes in Neisseria gonorrhoeae. Cell 47: 267–276. 2876777 10.1016/0092-8674(86)90449-6

[32] Marceau M, Beretti JL, Nassif X (1995) High adhesiveness of encapsulated Neisseria meningitidis to epithelial cells is associated with the formation of bundles of pili. Mol Microbiol 17: 855–863. 8596435 10.1111/j.1365-2958.1995.mmi_17050855.x

[33] Jennings MP, Virji M, Evans D, Foster V, Srikhanta YN, et al (1998) Identification of a novel gene involved in pilin glycosylation in Neisseria meningitidis. Mol Microbiol 29: 975–984. 9767566 10.1046/j.1365-2958.1998.00962.x

[34] Power PM, Roddam LF, Rutter K, Fitzpatrick SZ, Srikhanta YN, et al (2003) Genetic characterization of pilin glycosylation and phase variation in Neisseria meningitidis. Mol Microbiol 49: 833–847. 12864863 10.1046/j.1365-2958.2003.03602.x

[35] Borud B, Viburiene R, Hartley MD, Paulsen BS, Egge-Jacobsen W, et al (2011) Genetic and molecular analyses reveal an evolutionary trajectory for glycan synthesis in a bacterial protein glycosylation system. Proc Natl Acad Sci U S A 108: 9643–9648. 21606362 10.1073/pnas.1103321108 PMC3111294

[36] Haas R, Schwarz H, Meyer TF (1987) Release of soluble pilin antigen coupled with gene conversion in Neisseria gonorrhoeae. Proc Natl Acad Sci U S A 84: 9079–9083. 2892194 10.1073/pnas.84.24.9079 PMC299695

[37] Bayliss CD, Hoe JC, Makepeace K, Martin P, Hood DW, et al (2008) Neisseria meningitidis escape from the bactericidal activity of a monoclonal antibody is mediated by phase variation of lgtG and enhanced by a mutator phenotype. Infect Immun 76: 5038–5048. 18694967 10.1128/IAI.00395-08 PMC2573340

[38] Richardson AR, Yu Z, Popovic T, Stojiljkovic I (2002) Mutator clones of Neisseria meningitidis in epidemic serogroup A disease. Proc Natl Acad Sci U S A 99: 6103–6107. 11983903 10.1073/pnas.092568699 PMC122909

[39] Zhu P, Klutch MJ, Bash MC, Tsang RS, Ng LK, et al (2002) Genetic diversity of three lgt loci for biosynthesis of lipooligosaccharide (LOS) in Neisseria species. Microbiology 148: 1833–1844. 12055303 10.1099/00221287-148-6-1833

[40] Zhu P, Morelli G, Achtman M (1999) The opcA and (psi)opcB regions in Neisseria: genes, pseudogenes, deletions, insertion elements and DNA islands. Mol Microbiol 33: 635–650. 10417653 10.1046/j.1365-2958.1999.01514.x

[41] Keiser PB, Biggs-Cicatelli S, Moran EE, Schmiel DH, Pinto VB, et al (2011) A phase 1 study of a meningococcal native outer membrane vesicle vaccine made from a group B strain with deleted lpxL1 and synX, over-expressed factor H binding protein, two PorAs and stabilized OpcA expression. Vaccine 29: 1413–1420. 21199704 10.1016/j.vaccine.2010.12.039

[42] Singh B, Su YC, Riesbeck K (2010) Vitronectin in bacterial pathogenesis: a host protein used in complement escape and cellular invasion. Mol Microbiol 78: 545–560. 20807208 10.1111/j.1365-2958.2010.07373.x

[43] Singh B, Blom AM, Unal C, Nilson B, Morgelin M, et al (2010) Vitronectin binds to the head region of Moraxella catarrhalis ubiquitous surface protein A2 and confers complement-inhibitory activity. Mol Microbiol 75: 1426–1444. 20199596 10.1111/j.1365-2958.2010.07066.x

[44] Schmiel DH, Moran EE, Keiser PB, Brandt BL, Zollinger WD (2011) Importance of Antibodies to Lipopolysaccharide in Natural and Vaccine-Induced Serum Bactericidal Activity against Neisseria meningitidis Group B. Infect Immun 79: 4146–4156 IAI.05125-11 [pii];10.1128/IAI.05125-11 [doi]. 21768280 10.1128/IAI.05125-11 PMC3187254

[45] Ram S, Sharma AK, Simpson SD, Gulati S, McQuillen DP, et al (1998) A novel sialic acid binding site on factor H mediates serum resistance of sialylated Neisseria gonorrhoeae. J Exp Med 187: 743–752. 9480984 10.1084/jem.187.5.743 PMC2212180

[46] Wakarchuk WW, Gilbert M, Martin A, Wu Y, Brisson JR, et al (1998) Structure of an alpha-2,6-sialylated lipooligosaccharide from Neisseria meningitidis immunotype L1. Eur J Biochem 254: 626–633. 9688275 10.1046/j.1432-1327.1998.2540626.x

[47] Jones DM, Borrow R, Fox AJ, Gray S, Cartwright KA, et al (1992) The lipooligosaccharide immunotype as a virulence determinant in Neisseria meningitidis. Microb Pathog 13: 219–224. 1283998 10.1016/0882-4010(92)90022-g

[48] Rytkonen A, Albiger B, Hansson-Palo P, Kallstrom H, Olcen P, et al (2004) Neisseria meningitidis undergoes PilC phase variation and PilE sequence variation during invasive disease. J Infect Dis 189: 402–409. 14745697 10.1086/381271

[49] Criss AK, Bonney KM, Chang RA, Duffin PM, LeCuyer BE, et al (2010) Mismatch correction modulates mutation frequency and pilus phase and antigenic variation in Neisseria gonorrhoeae. J Bacteriol 192: 316–325. 19854909 10.1128/JB.01228-09 PMC2798252

[50] Hagblom P, Segal E, Billyard E, So M (1985) Intragenic recombination leads to pilus antigenic variation in Neisseria gonorrhoeae. Nature 315: 156–158. 2859529 10.1038/315156a0

[51] Craig L, Volkmann N, Arvai AS, Pique ME, Yeager M, et al (2006) Type IV pilus structure by cryo-electron microscopy and crystallography: implications for pilus assembly and functions. Mol Cell 23: 651–662. 16949362 10.1016/j.molcel.2006.07.004

[52] Parge HE, Forest KT, Hickey MJ, Christensen DA, Getzoff ED, et al (1995) Structure of the fibre-forming protein pilin at 2.6 A resolution. Nature 378: 32–38. 7477282 10.1038/378032a0

[53] Blom AM, Rytkonen A, Vasquez P, Lindahl G, Dahlback B, et al (2001) A novel interaction between type IV pili of Neisseria gonorrhoeae and the human complement regulator C4B-binding protein. J Immunol 166: 6764–6770. 11359834 10.4049/jimmunol.166.11.6764

[54] Kahler CM, Martin LE, Tzeng YL, Miller YK, Sharkey K, et al (2001) Polymorphisms in pilin glycosylation Locus of Neisseria meningitidis expressing class II pili. Infect Immun 69: 3597–3604. 11349019 10.1128/IAI.69.6.3597-3604.2001 PMC98345

[55] Chamot-Rooke J, Mikaty G, Malosse C, Soyer M, Dumont A, et al (2011) Posttranslational modification of pili upon cell contact triggers N. meningitidis dissemination. Science 331: 778–782. 21311024 10.1126/science.1200729

[56] Helaine S, Carbonnelle E, Prouvensier L, Beretti JL, Nassif X, et al (2005) PilX, a pilus-associated protein essential for bacterial aggregation, is a key to pilus-facilitated attachment of Neisseria meningitidis to human cells. Mol Microbiol 55: 65–77. 15612917 10.1111/j.1365-2958.2004.04372.x

[57] Lappann M, Haagensen JA, Claus H, Vogel U, Molin S (2006) Meningococcal biofilm formation: structure, development and phenotypes in a standardized continuous flow system. Mol Microbiol 62: 1292–1309. 17121595 10.1111/j.1365-2958.2006.05448.x

[58] Chiang SL, Taylor RK, Koomey M, Mekalanos JJ (1995) Single amino acid substitutions in the N-terminus of Vibrio cholerae TcpA affect colonization, autoagglutination, and serum resistance. Mol Microbiol 17: 1133–1142. 8594332 10.1111/j.1365-2958.1995.mmi_17061133.x

[59] Weiser JN, Pan N, McGowan KL, Musher D, Martin A, et al (1998) Phosphorylcholine on the lipopolysaccharide of Haemophilus influenzae contributes to persistence in the respiratory tract and sensitivity to serum killing mediated by C-reactive protein. J Exp Med 187: 631–640. 9463413 10.1084/jem.187.4.631 PMC2212159

[60] Volanakis JE (2001) Human C-reactive protein: expression, structure, and function. Mol Immunol 38: 189–197. 11532280 10.1016/s0161-5890(01)00042-6

[61] Casey R, Newcombe J, McFadden J, Bodman-Smith KB (2008) The acute-phase reactant C-reactive protein binds to phosphorylcholine-expressing Neisseria meningitidis and increases uptake by human phagocytes. Infect Immun 76: 1298–1304. 18195032 10.1128/IAI.00741-07 PMC2258818

[62] Vogel U, Claus H, Heinze G, Frosch M (1997) Functional characterization of an isogenic meningococcal α-2,3- sialyltransferase mutant: the role of lipooligosaccharide for serum resistance in serogroup B meningococci. Med Microbiol Immunol Berl 186: 159–166. 9403845 10.1007/s004300050059

[63] Madico G, Welsch JA, Lewis LA, McNaughton A, Perlman DH, et al (2006) The meningococcal vaccine candidate GNA1870 binds the complement regulatory protein factor H and enhances serum resistance. J Immunol 177: 501–510. 16785547 10.4049/jimmunol.177.1.501 PMC2248442

[64] Unkmeir A, Latsch K, Dietrich G, Wintermeyer E, Schinke B, et al (2002) Fibronectin mediates Opc-dependent internalization of Neisseria meningitidis in human brain microvascular endothelial cells. Mol Microbiol 46: 933–946. 12421301 10.1046/j.1365-2958.2002.03222.x

[65] Ram S, Cox AD, Wright JC, Vogel U, Getzlaff S, et al (2003) Neisserial lipooligosaccharide is a target for complement component C4b. Inner core phosphoethanolamine residues define C4b linkage specificity. J Biol Chem 278: 50853–50862. 14525973 10.1074/jbc.M308364200

[66] Merker P, Tommassen J, Kusecek B, Virji M, Sesardic D, et al (1997) Two-dimensional structure of the Opc invasin from Neisseria meningitidis. Mol Microbiol 23: 281–293. 9044262 10.1046/j.1365-2958.1997.2051567.x

[67] Scholten RJ, Poolman JT, Valkenburg HA, Bijlmer HA, Dankert J, et al (1994) Phenotypic and genotypic changes in a new clone complex of Neisseria meningitidis causing disease in The Netherlands, 1958–1990. J Infect Dis 169: 673–676. 8158049 10.1093/infdis/169.3.673

[68] Apicella MA, Griffiss JM, Schneider H (1994) Isolation and characterization of lipopolysaccharides, lipooligosaccharides, and lipid A. Methods Enzymol 235: 242–252. 8057898 10.1016/0076-6879(94)35145-7

[69] Zollinger WD, Mandrell RE (1977) Outer-membrane protein and lipopolysaccharide serotyping of Neisseria meningitidis by inhibition of a solid-phase radioimmunoassay. Infect Immun 18: 424–433. 72735 10.1128/iai.18.2.424-433.1977 PMC421250

[70] Dunn KL, Virji M, Moxon ER (1995) Investigations into the molecular basis of meningococcal toxicity for human endothelial and epithelial cells: the synergistic effect of LPS and pili. Microb Pathog 18: 81–96. 7643745 10.1016/s0882-4010(95)90085-3

[71] Kurzai O, Schmitt C, Claus H, Vogel U, Frosch M, et al (2005) Carbohydrate composition of meningococcal lipopolysaccharide modulates the interaction of Neisseria meningitidis with human dendritic cells. Cell Microbiol 7: 1319–1334. 16098219 10.1111/j.1462-5822.2005.00559.x

